# Identification and validation of synergistic drug strategies targeting macrophage polarization in triple-negative breast cancer via single-cell transcriptomics and deep learning

**DOI:** 10.1016/j.tranon.2025.102457

**Published:** 2025-06-27

**Authors:** Qi Qi, Wenhao Yang, Liang Li, Yuheng Tang, Yongzhi Chen, Hui Wang, Sun Yingjie, Jialin Shi, Samina Gul, Wenru Tang, Jianyu Pang, Xiaoli Xie

**Affiliations:** Laboratory of Molecular Genetics of Aging & Tumor, Medicine School, Kunming University of Science and Technology, China

**Keywords:** Triple-negative breast cancer, Tumor microenvironment, Macrophage polarization, ZBTB20, Combination therapy

## Abstract

Triple-negative breast cancer (TNBC) presents therapeutic challenges due to its immunosuppressive tumor microenvironment (TME) and limited targeted options. Analyzing scRNA-seq data from 24 TNBC patients using integrated transcriptomics, machine-learning, and pseudo-time trajectory mapping, we developed a macrophage differentiation-based classifier (MMDCSS) (CD93, CHI3L1, ZBTB20) with remarkable prognostic accuracy (C-index: 0.929, 3-year AUC: 0.907). Computational pharmacology identified finasteride as a ZBTB20 modulator (binding energy:7.7 kcal/mol) capable of reversing tumor-induced M2 macrophage polarization. Finasteride significantly enhanced doxorubicin efficacy ex vivo and in vivo by reprogramming the TME, reducing M2 macrophage infiltration while promoting an M1 phenotype. Mechanistically, finasteride upregulated ZBTB20, counteracting TNBC-mediated suppression of this M1-associated transcription factor. Our findings establish ZBTB20 as a key regulator of macrophage polarization in TNBC and introduce finasteride as a clinically viable agent to reverse TME immunosuppression. Targeting macrophage polarization via ZBTB20 modulation, particularly by repurposing finasteride, offers a promising therapeutic strategy for TNBC with immediate translational potential.

## Highlights

• A novel macrophage differentiation-based classifier (MMDCSS) accurately predicts prognosis in TNBC patients.
• Repurposing finasteride to modulate ZBTB20 offers a clinically viable strategy to reverse immunosuppression and improve TNBC therapy.

**List of abbreviations**
TNBC: Triple-negative breast cancer;
scRNA-seq: Single Cell RNA sequencing;
MMDCSS: M1/M2 macrophage differentiation characteristics scoring system;
TPA: Tetradecanoylphorbol Acetate;
TIME: tumor immune microenvironment;
TAM: tumor-associated macrophages;
CTD: Comparative Toxicogenomics Database;
DEGs: differential genes;
TIGS: tumor immunogenicity score;
TMB: tumor mutational burden;
APM: antigen processing presentation mechanism;
KEGG: Kyoto Encyclopedia of Genes and Genomes;
GO: Gene Ontology;
Treg: Regulatory T cell;
NK: Natural killer cell;
NKT: Natural killer T cell;
C-index: concordance index.

## Introduction

Triple-negative breast cancer (TNBC) represents one of the most formidable challenges in cancer therapeutics, accounting for approximately 10 % of breast cancer cases but responsible for a disproportionately high number of cancer-related deaths [[1], [2], [3]]. The aggressive nature of TNBC, characterized by frequent p53 mutations, rapid proliferation, and high metastatic potential, coupled with the absence of targetable receptors (estrogen, progesterone, and HER2), severely limits therapeutic options and contributes to poor clinical outcomes [1,2]. Despite advances in neoadjuvant chemotherapy, response rates remain suboptimal, with 5-year survival rates significantly lower than other breast cancer subtypes [4,5]. This critical unmet clinical need demands innovative approaches that transcend conventional treatment paradigms and address the fundamental biological mechanisms driving TNBC progression.

Emerging evidence has established the tumor immune microenvironment (TIME) as a critical determinant of cancer progression and therapeutic response, with macrophages emerging as pivotal orchestrators of this complex ecosystem [6,7]. Macrophages exhibit remarkable plasticity, polarizing along a spectrum between classical pro-inflammatory M1 phenotypes with anti-tumor activity and alternative M2 phenotypes that promote tumor growth, angiogenesis, and immunosuppression [[8], [9], [10]]. In TNBC, tumor-associated macrophages (TAMs) predominantly display M2-like characteristics and correlate with poor prognosis, therapy resistance, and metastatic dissemination [11,12]. On the other hand, it has been shown that damage-associated molecular patterns (DAMPs, e.g. HMGB1, ATP) released from necrotic regions within the tumor can induce macrophage polarisation towards the M2 phenotype via the TLR4 pathway. Therefore, the aggregation of M2 macrophages may be a consequence of tumor necrosis rather than a direct pro-tumor cause [13].

The dynamic interplay between cancer cells and TAMs, mediated through complex networks of cytokines, metabolites, and signaling molecules, creates a self-reinforcing immunosuppressive microenvironment that shields tumors from immune surveillance [[14], [15], [16]]. Despite the recognized importance of macrophage polarization in TNBC progression, the molecular mechanisms governing this process remain incompletely understood, and therapeutic strategies precisely targeting these pathways are notably lacking [17,18].

The convergence of single-cell RNA sequencing (scRNA-seq) and machine learning has revolutionized our ability to decode the complex cellular heterogeneity and molecular networks within the tumor microenvironment [19,20]. When integrated with advanced machine learning algorithms, this technology enables the identification of previously unrecognized cellular signatures and regulatory factors with profound prognostic and therapeutic implications [[21], [22], [23]]. However, translating these computational insights into effective therapeutic strategies for TNBC remains a significant challenge, particularly in developing targeted approaches to reprogram the immunosuppressive tumor microenvironment [24].

In this study, we address this critical gap by developing an integrated computational-experimental framework to identify and therapeutically target key regulators of macrophage polarization in TNBC. By analyzing scRNA-seq data from 24 TNBC patients through pseudotime trajectory analysis, we uncovered the molecular dynamics of macrophage polarization and constructed a machine learning-based M1/M2 Macrophage Differentiation Characteristics Scoring System (MMDCSS) with remarkable prognostic value (C-index: 0.929). This analysis revealed ZBTB20 as a previously unrecognized master regulator of M1 macrophage polarization. Through virtual molecular docking and deep learning-based drug synergy prediction, we identified finasteride as a novel ZBTB20-targeting agent capable of reversing tumor-induced M2 polarization. Significantly, finasteride synergistically enhanced the anti-tumor efficacy of doxorubicin by reprogramming the immunosuppressive microenvironment, as validated in both in vitro and in vivo models. Our findings not only provide mechanistic insights into the molecular regulation of macrophage polarization in TNBC but also establish a promising therapeutic strategy through drug repurposing that could be rapidly translated into clinical practice.

## Materials and methods

### Data sources

The scRNA-seq queue is downloaded from the GEO database with index numbers GSE143423, GSE176078, GSE148673, and GSE161529. The Bulk RNA-seq queue is downloaded from the TCGA and GEO databases, respectively, where the TCGA-BRCA queue is obtained from UCSC XENA, and the verification queues GSE58812 and GSE173839 are downloaded from the GEO database.

### The scRNA-seq data analysis

Standard analytical procedures were performed for four scRNA-seq cohorts based on Seurat [25]. The quality control standards were set as min.cells = 3, min.features = 200, and percent.mt < 10. After the dimension reduction of PCA, the Harmony [26] is used to integrate the four scRNA-seq matrices and remove the batch effect. In low-resolution analysis, Top30 PCs were selected as the input of UMAP dimensionality reduction, and resolution=0.4 was selected as the standard of cell clustering. In the high-resolution analysis, the parameters used in UMAP dimensionality reduction and SNN cell clustering of different subsets are also different. For immune cell subsets, select dim=30, resolution=0.6; for macrophage subsets, select dim=30, resolution=0.5. To annotate the cell clusters in a semi-supervised way, we first automatically predict the cell clusters based on SingleR [27], then find the Marker gene (minpct=0.5, logfcthreshold=0.5) of each cluster according to the FindAllMarker function, and query the specific information of the Marker gene based on the CellMaker database [28]. Finally, the cell clusters were evaluated by combining the results of automatic annotation and Marker gene annotation.

### Pseudo-time analysis of subgroups

As a popular algorithm of pseudo-time analysis in scRNA-seq advanced analysis, Monocle [29,30] is widely used in scRNA-seq data research. Like Seurat, Monocle can reduce and cluster the single-cell matrix, except that it can evaluate the differentiation status of cells according to the genetic information of cell clusters. In our study, the highly variant genes calculated by Monocle (mean_expression>=0.1, dispersion_empirical>=1*dispersion_fit) were selected as the input for pseudo-time axis calculation. DDRTree was used to reduce the dimension of cell cluster information (reduceDimension=2). On the pseudo-time axis, 0–20 represents the differentiation state, where higher values indicate closer proximity to the terminal differentiation stage. Additionally, characteristic genes associated with M1 and M2 macrophage differentiation were selected based on the parameters minpct = 0.1 and logfcthreshold = 0.25. ClusterProfiler [31] was used to explore the enrichment of GSEA pathway activity of M1/M2 differentiation characteristic genes.

### Construction and verification of machine learning model

Elastic Network is one of the machine learning algorithms that makes the model more stable and sensitive by controlling the regularization of L1 and L2. Before constructing the machine learning model, DEseq2 was used to screen the differential genes (DEGs) of TNBC patients, and the screening threshold was *p* < 0.05, |logFC|=1. In the following study, caret was used to calculate the best L1 and L2 hyperparameters, the number of iterations was 100, and 500 random seeds were set to screen the best results. In the training process of the Elastic Network model, 10-fold cross-validation is used to verify the training model. In order to ensure that the final machine learning model accords with the prediction of prognosis, we again use Multivariate Cox regression analysis to screen the variables trained by the Elastic Network and finally use the regression coefficient of Multivariate Cox as the model's coefficient. In addition, the patients' MMDCSS was grouped according to the median and then analyzed by KM survival curve to compare the difference in survival rate between high and low levels of MMDCSS.

### Evaluation of clinical application value

MMDCSS was included in the characteristic clinical cohort. Univariate Cox and Multivariate Cox regression were performed to evaluate whether MMDCSS is clinically independent and a risk factor according to HR. Under the premise of *p* < 0. 05, HR>1 indicates that the variable is a risk factor, and HR<1 indicates that it is a protective factor. In addition, the Nomogram of MMDCSS and its calibration curve are constructed using rms. The ESTIMATE algorithm is used to evaluate the relationship between the immune and stromal components of TIME and MMDCSS. Subsequently, tumor immunogenicity score (TIGS) was used to evaluate the relationship between MMDCSS and immunotherapy response. This index combines tumor mutational burden (TMB) and antigen processing presentation mechanism (APM) (APMnormalized × ln (TMB+1)) to predict immune response, which further improves the accuracy of this index and is the key index to determine whether to respond to immunotherapy in the clinic [32]. The higher the TIGS, the stronger the immune response ability of the patient, and the immunotherapy received is more likely to have a targeted effect.

### Research on prognostic mechanism of MMDCSS

Studying the components of MMDCSS alone is helpful to understand further the adverse prognostic reactions caused by MMDCSS. The expression differences of MMDCSS components in different cell types and patients in scRNA-seq and Bulk RNA-seq were examined, respectively, and their results in tumor/normal tissues were verified by the HPA database [33]. Next, the top 30 % of the constituent gene expression of MMDCSS was defined as the high-expression group, and the last 70 % was defined as the low-expression group. The DEGs between the two groups were screened, and the pathway enrichment analysis was carried out. After screening the significantly enriched pathway, the pathway's activity was further calculated by GSVA. Finally, the activity differences of pathways between high and low-expression groups were compared to explore the specific molecular mechanism of prognosis. The DEGs of internal genes in MMDCSS were screened by limma [34] (*p* < 0.05, |log2FC|=1), and the ClusterProfiler was enriched by the Kyoto Encyclopedia of Genes and Genomes (KEGG) or Gene Ontology (GO) pathway.

### Prediction of synergy between virtual docking of targeted drugs and deep learning

First, the targeting antineoplastic drugs of internal genes in MMDCSS are screened based on the CTD [35]. Then, through the 3D structure of the drug list in the PubChem database [36], the 3D structure of the protein encoded by the gene was downloaded from the Uniport database [37]. Next, the batch molecular docking is carried out by Autodock [38], and the final targeted drug (Docking energy<−1 kcal/mol) is screened according to the docking energy. The lower the energy is, the less energy is needed for the combination of the two. That is, the more likely to produce targeted interaction. In addition, highly toxic substances and harmful drugs are excluded according to previous reports and the toxicological characteristics of drugs. Finally, the Deep Learning model was used to predict the synergism between different combinations of targeted drugs. Subsequently, the open-source version of pymol was used to visualize the docking results between drugs and proteins. The Deep Learning framework was used for model training based on the synergistic values of 38 drug combinations among 39 cell lines measured in the experiment. First of all, based on the smile structure of each drug, rdkit is used to transform it into a molecular fingerprint, and 2048 features encode each fingerprint. Cell lines were obtained by expression profile, and each cell line was composed of 3985 characteristics. Then, taking the drug and cell line matrix as input items through tensorflow, a 5-layer depth network was set up for 1000 iterations. The training parameters were as follows: act_func=tf.nn.relu, dropout=0.5, input_dropout=0.2, eta=0.00001, norm='tanh', finally obtained a stable prediction model of the synergistic effect of combined drug use [39]. Finally, the model was used to predict the combination of various targeted drugs screened in this study.

### Statistical analysis

This study mainly uses the R 4.1.1 version for statistical analysis. Wilcox test was used for an inter-group statistical test, Chi-Squared was used to test the significant significance of M1/M2 differentiation-related gene mutation probability, and a log-rank test was used to test the difference of survival probability between samples. *P* < 0.05 was statistically significant.

### Cell culture

After rapidly thawing the frozen cell storage tubes, cells were centrifuged to remove the cryopreservation solution containing 10 % DMSO. The cells were then resuspended in a preheated medium and inoculated for culture. THP-1 cells were subcultured in 1640 medium, maintaining cell density through medium replenishment or partial medium exchange. Adherent cells were washed with 1 % PBS, digested with 0.25 % trypsin, neutralized with Gibco DMEM containing 8 % FBS, and then subcultured proportionally. For cell cryopreservation, a cryopreservation solution containing 10 % DMSO was prepared, and cells were stored in liquid nitrogen after programmed cooling.

### Induction of differentiation and drug administration

THP-1 cells were seeded into 6-well plates at 1.5 × 10^6^ cells/well in 1640 complete medium containing 100 ng/mL TPA (2 mL/well). The cells were incubated at 37 °C and 5 % CO2 for 24 h to allow adherence. The medium was then discarded, and the cells were washed three times with PBS before being cultured in a fresh 1640 medium for another 24 h. Subsequently, for M1-type induction, LPS (100 ng/mL) and IFN-γ (20 ng/mL) were added, while for M2-type induction, IL-4 and IL-13 (20 ng/mL each) were added. After 24 h of treatment, the medium was replaced with a normal medium. The working concentration of finasteride was 10 μM.

### Cell co-culture

THP-1 cells were differentiated and adherent as described above and set aside. When TNBC cells reached 80 % confluence, they were cultured in a DMEM medium containing 0.1 % serum for 24 h. The supernatant was then collected, filtered through a 0.22 μm membrane, and stored at −80 °C (avoiding repeated freezing and thawing). 1.37 mL of TNBC supernatant was mixed with 130 μL of serum (final concentration 8 %) and added to the THP-1 cell wells. The 1640 complete medium was added to a total volume of 1.5 mL/well, and the cells were co-cultured for observation. Key parameters included TPA-induced adherence, serum gradient regulation, and sterile filtration for preservation.

### CCK-8 drug toxicity assay

THP-1 cells were seeded into 96-well plates at 5 × 10^4^ cells/well in a medium containing 100 ng/mL TPA (100 μL/well). The cells were incubated at 37 °C and 5 % CO2 for 24 h to induce adherence. The medium was then discarded, and the cells were washed three times with PBS before being cultured in a normal medium for another 24 h. A concentration gradient of MK-906 drug was set up (≥5 replicates per group, including a blank control), and 100 μL of drug mixture (diluted from a 200 mM stock solution) was added to each well. The cells were incubated for another 24 h. Subsequently, a 10 % volume of CCK-8 reagent was added in the dark, and the cells were incubated for 30 min. Absorbance at 450 nm and 650 nm was measured using a microplate reader (calculating ΔOD = OD450 - OD650). Measurements were taken every 30 min until the value reached around 1.

### Establishment of a subcutaneous tumor model in mice

The experiment used female BALB/c mice aged 6 to 8 weeks from Henan Skobes Biotechnology Co., Ltd. 4T1 cells were first thawed, centrifuged, and resuspended in PBS and a complete medium for culture and subculture. When preparing the injection solution, the Matrigel was placed in a 4 °C refrigerator to melt. Each site contains 5 × 10^5^ cells dissolved in 80 μL of injection solution. The cells were resuspended in pre-cooled FBS and mixed with the Matrigel at a 1:1 ratio while keeping the temperature low. Before injection, the lower abdominal hair of the mice was shaved using a shaving device, and depilatory cream was applied and then wiped off. The mice were anesthetized with approximately 50 μL of 3 % pentobarbital sodium, and a pre-cooled syringe was used to aspirate the cell suspension. After discharging any air bubbles, the cells were injected subcutaneously into the mammary gland area of the mice's abdomen. After the injection, the mice were grouped and ear-tagged after waking up. Their condition was observed 6 h later. Drug administration began after tumor formation, with MK-906 administered at 0.5 mg/kg by gavage and ADR at 2 mg/kg by intraperitoneal injection. Tumor volume and mouse body weight were measured every three days after tumor formation. During tissue collection, the mice were anesthetized with isoflurane and euthanized by cervical dislocation. The tumors were then weighed and processed for subsequent experiments. All animal procedures were approved by the IACUC of Kunming University of Science and Technology.

### Flow cytometry analysis

Approximately 40 mg of tumor tissue was cut into small pieces and incubated with 400 μL of tissue digestion solution at 37 °C and 180 rpm/min for 1–2 h until the tissue fragments were lysed. The reaction was terminated with DMEM containing 8 % serum, filtered through a 300-mesh cloth, and centrifuged at 1200 rpm/min for 5 min. The supernatant was discarded, and the cells were washed once with DMEM before being resuspended in a suitable concentration of medium. For cell surface staining (CD45 CD11b), the cells were first incubated with a live/dead dye at room temperature in the dark for 20 min. The reaction was then terminated and washed with serum-containing DMEM and 1x PBS, followed by centrifugation and supernatant removal. After blocking, the cells were incubated with surface antibodies for 30 min, washed, resuspended in 1 % PFA, and stored until analysis. For intracellular staining, the surface staining steps were completed first, followed by incubation with membrane fixation and permeabilization solution for 45 min and staining with intracellular antibodies (CD86 CD206) for 30 min. The cells were then washed with membrane wash solution, resuspended in PBS, and stored until analysis. Fluorescent antibodies for flow cytometry were obtained from BioLegend.

### Western blot

For protein extraction, treated cells were placed on ice, washed three times with pre-cooled PBS, and lysed with RIPA lysis buffer containing PMSF on ice for 10 min. The cells were then scraped and transferred to an EP tube. After ultrasonic fragmentation, the lysate was centrifuged at 4 °C and 13,000 rpm/min for 20 min. The supernatant was collected to measure protein concentration or stored at −80 °C. 200 mM PMSF was prepared by mixing 10 mL of isopropanol with 0.348 g of PMSF and stored in aliquots at −20 °C.

For protein quantification, BCA standards were prepared, and the B and A solutions were mixed at a 1:50 ratio. The mixture was added to a 96-well plate and incubated at 37 °C for 30 min. Absorbance at 562 nm was measured, and a standard curve was created to calculate protein concentration and the loading system. The protein sample system consisted of 4 μL of 5 × SDS PAGE dye, X μL of protein sample, (10 - X) μL of 1 × PBS and a total volume of 20 μL. The 5 × SDS PAGE dye formula consisted of 1.55 mL of 1 M Tris–HCl (pH=6.8), 0.5 g of SDS, 5 mg of bromophenol blue, 2.5 mL of glycerol, and ddH2O to a total volume of 10 mL. The dye was stored at room temperature and β-mercaptoethanol was added at a 1:19 ratio before use.

For SDS-PAGE gel electrophoresis, glass plates were fixed, and the system was leak-tested with ddH2O before pouring and solidifying the gel. 10 % lower and upper gels were prepared and poured into the glass plates, and a comb was inserted to solidify. SDS-PAGE Running Buffer was added, the comb was removed, samples and protein markers were loaded, and electrophoresis was performed at a constant current of 30 mA per gel for approximately 1 hour and 40 min. During the transfer, ice water and 1 × Transfering Buffer were prepared, the PVDF membrane was activated, and a "sandwich" structure was created for transfer at 200 mA and 300 V for 2 h. After transfer, the membrane was washed with 1 × TBST for 5 min, blocked with blocking solution for 2 h or overnight at 4 °C, washed again, and the membrane was cut into strips. Primary antibodies were incubated overnight, secondary antibodies were incubated for 2 h, and the membrane was developed. If strip removal was necessary, the membrane was washed with TBST for 5 min, stripped with stripping buffer for 20 min, washed three times with TBST, blocked for 30 min, and then incubated with primary and secondary antibodies.

### Luminex assay

The experiment employed Luminex technology from the Serap company. The instrumentation consisted of a magnetic plate, a horizontal shaker (operating at 800±50 rpm), a centrifuge, pipettes with matching tips, double-distilled water, a 500 mL graduated cylinder, EP tubes, and 1xPBS. The reagent kit was removed from storage half an hour before use and allowed to equilibrate at room temperature. Samples were dissolved, mixed uniformly, and centrifuged at 4 °C. Microbeads were subjected to ultrasonication for 30 s, followed by shaking for 1 min. Then, 60 mL of the microbeads were transferred to a mixing vial and diluted to a final volume of 3 mL. Standard substances were dissolved in 100 μL of diluent and serially diluted. 50 μL of both the standard substances and the samples were added to their respective wells, followed by the addition of 50 μL of the bead mixture. The plate was incubated at room temperature for 2 h. The plate was washed three times, using 100 μL of wash solution per well each time. 50 μL of detection antibody was added to each well, and the plate was incubated with shaking in the dark at room temperature for 1 hour. 50 μL of SAPE was added to each well, and the plate was incubated with shaking in the dark at room temperature for 30 min. The plate was washed three times again, using 200 μL of wash solution per well. 100 μL of sheath fluid was added to each well, and the plate was incubated with shaking in the dark at room temperature for 2 min. Finally, the plate was analyzed using the Luminex instrument, and the data were exported and analyzed.

## Results

### Imbalance of macrophage polarization in the tumor microenvironment of triple-negative breast cancer

**Fig. 1:**
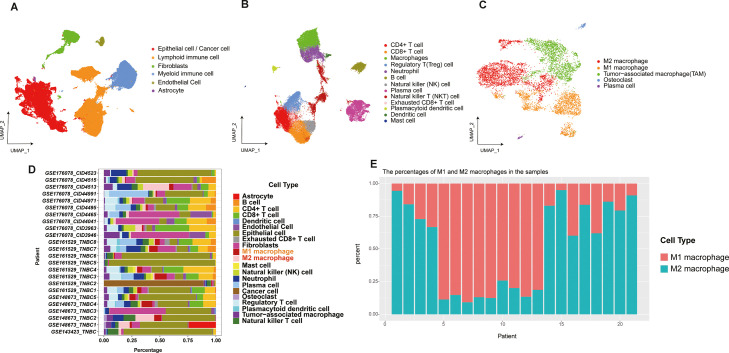
Integrated analysis of single-cell RNA sequencing (scRNA-seq) data from 24 triple-negative breast cancer (TNBC) patients. **A.** UMAP plot of overall cell clustering in the TNBC microenvironment. **B.** Re-clustering of immune cell subpopulations. **C.** Re-clustering and annotation of macrophage subpopulations. **D-E.** Distribution of M1/M2 macrophage ratios in patient samples.

By integrating four scRNA-seq cohorts with the Harmony algorithm, we obtained information on 90,472 cells, and these cells were initially partitioned into 33 cell clusters at the initial clustering level. Based on this, we constructed a high-resolution cellular atlas of the triple-negative breast cancer (TNBC) tumor microenvironment. In the second-level annotation, six major cell types were identified, including epithelial/cancer cells, lymphoid immune cells, fibroblasts, myeloid immune cells, endothelial cells, and astrocytes (Fig. 1A). To further explore cellular details, we initially sub-classified lymphoid and myeloid immune cells. At high resolution, the immune cells were delineated into 13 distinct immune cell types (Fig. 1B).

Macrophages, which have garnered significant attention in previous reports, underwent detailed downstream analysis to reveal additional insights. Within our integrated data, the high-resolution macrophage subpopulations were further annotated and subdivided into five cell information types, M2 macrophages, M1 macrophages, tumor-associated macrophages (TAMs), osteoclasts, and plasma cells (Fig. 1C).

It is worth noting that the analysis of macrophage subpopulations revealed a generally higher proportion of M2 macrophages compared to M1 macrophages in the microenvironment of most triple-negative breast cancer (TNBC) patients (Fig. 1D), and macrophages occupied a significant proportion in the tumor microenvironment (Fig. 1E).

In contrast, the higher content of M2 macrophages compared to M1 macrophages in patient samples suggests a pivotal role for M2 macrophages in the tumor microenvironment (TIME). This polarization imbalance is closely associated with immune evasion and poor prognosis in triple-negative breast cancer.

Furthermore, we conducted an in-depth investigation of different cell groups within the tumor microenvironment (TIME), all of which exhibited specific genetic information. The characteristic markers of the corresponding cells are presented in Supplementary Figure S1. (Figure S1)

### Molecular characteristics of macrophage polarization based on pseudo-time trajectory analysis and construction of a prognostic model (MMDCSS)

**Fig. 2:**
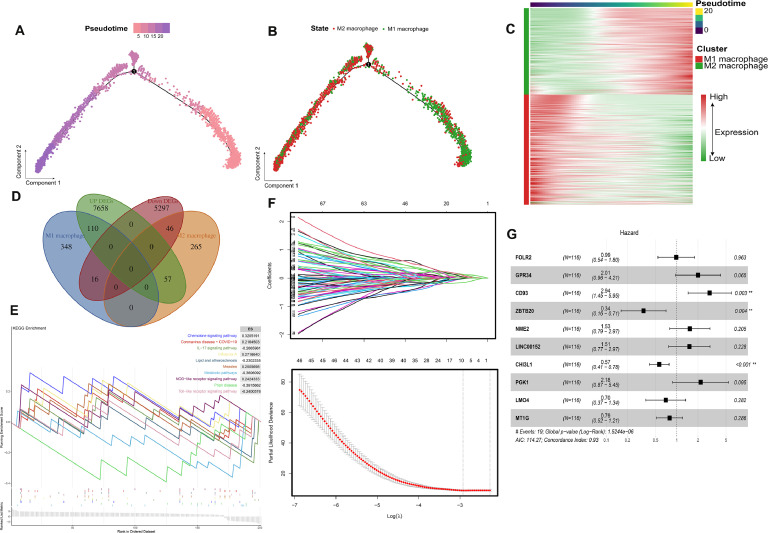
Molecular characteristics of macrophage polarization and construction of the MMDCSS prognostic model. **A.** Pseudo-temporal differentiation trajectory analysis of macrophages. **B.** Pseudo-temporal trajectory cluster diagram showing differentiation states. **C.** Dynamic expression changes of M1/M2 characteristic genes along the pseudo-temporal axis. **D.** Intersection analysis of macrophage polarization characteristic genes and differentially expressed genes in TNBC. **E.** GSEA functional enrichment results for macrophage polarization characteristic genes. **F.** Elastic net algorithm, after 100 iterations and 10-fold cross-validation, to screen prognostic characteristic genes. **G.** MMDCSS model constructed by multivariable Cox regression.

Based on single-cell transcriptome data from 24 TNBC patients, we employed pseudo-time analysis techniques to elucidate the developmental trajectory of macrophage polarization. The results indicated that M1 macrophages were located in the early stages of differentiation (Fig. 2A), whereas M2 macrophages were found in the later stages (Fig. 2B), suggesting a potential unidirectional polarization process from M1 to M2 in the TNBC microenvironment. By analyzing dynamic changes in gene expression along the polarization trajectory (Fig. 2C), we identified a series of genes with temporally specific expression patterns. Characteristic genes in the M1 macrophage cluster showed decreasing expression over time, while those in the M2 cluster demonstrated increasing expression.

To further delineate the differentiation features of M1 and M2 macrophages in TNBC, we intersected their differentiation genes with TNBC DEGs, resulting in a set of 229 candidate genes (Fig. 2D). Functional enrichment analysis of these genes uncovered coordinated changes in multiple signaling pathways during the M1/M2 polarization process. Notably, the activation of chemoattractant signaling and NOD-like receptor signaling, as well as the significant inhibition of metabolic pathways, were observed (Fig. 2E). This suggests that metabolic reprogramming may be a key mechanism driving the transition of macrophages from the anti-tumor M1 phenotype to the tumor-promoting M2 phenotype, providing new insights for targeted intervention in macrophage polarization.

Based on these findings, we utilized an elastic net algorithm to select ten prognostic markers from genes associated with macrophage polarization (Fig. 2F). Using multivariable Cox regression, we constructed the M1/M2 Macrophage Differentiation Characteristic Scoring System (MMDCSS): MMDCSS = 1.078*CD93–1.084*ZBTB20–0.570*CHI3L1 (Fig. 2G). This model exhibited excellent predictive performance in the development set (C-index: 0.929). The coefficient directions of the three core molecules were highly consistent with their biological functions in macrophage polarization: CD93 promotes M2 polarization (positive coefficient), ZBTB20 maintains the M1 phenotype (negative coefficient), and CHI3L1 regulates polarization shifts (negative coefficient). This model not only accurately predicts the prognosis of TNBC patients but also reveals key molecular targets for regulating macrophage polarization.

### Rigorous validation and comprehensive clinical application potential of the MMDCSS model

**Fig. 3:**
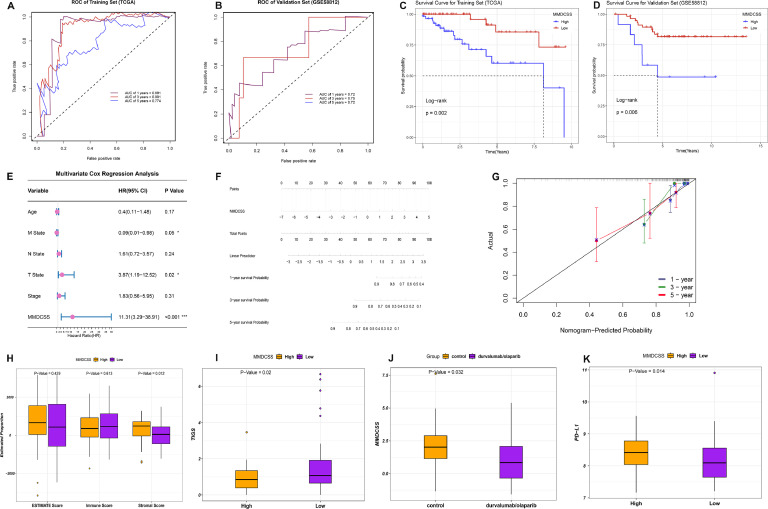
Validation of MMDCSS and Prediction of Immune Evaluation and Immunotherapy. **A.** ROC curves for MMDCSS in the training cohort and B. validation cohort. **C.** Kaplan-Meier survival curve for the training set, showing a significant correlation between high MMDCSS expression and poor prognosis. **D.** Kaplan-Meier survival curve for the external validation set. **E.** Multivariate Cox regression analysis revealing MMDCSS as an independent prognostic factor (HR=11.31). **F.** Clinical prediction nomogram integrating MMDCSS; G. Calibration Curve. **H.** Association between MMDCSS and the tumor immune microenvironment**. I.** TIGS analysis reveals an association between high MMDCSS expression and an immunosuppressive microenvironment. **J-K.** Analysis of immunotherapy cohorts and PD-L1 expression.

To comprehensively evaluate the clinical application potential of the MMDCSS model, we first rigorously validated its reliability using Biostatistics. In the training cohort, MMDCSS demonstrated excellent predictive performance. Specifically, the AUC value for 3-year survival prediction was as high as 0.901 (Fig. 3A). This outstanding performance was confirmed in an external independent validation cohort, with 1-year, 3-year, and 5-year AUCs all exceeding 0.7 (Fig. 3B), indicating the model's stability across cohorts and predictive durability over time. Kaplan-Meier survival analysis further showed that the survival rate of patients with high MMDCSS expression was significantly lower than that of patients with low expression (*P* < 0.001) (Fig. 3C-D), confirming the effectiveness of risk stratification based on macrophage polarization characteristics.

More importantly, multivariable Cox regression analysis revealed that MMDCSS is an independent risk factor for the prognosis of TNBC patients, with a hazard ratio (HR=11.31) much higher than traditional clinicopathological indicators (Fig. 3E). This suggests that molecular typing based on macrophage polarization characteristics may be superior to traditional typing methods. Based on this, we constructed a clinical prediction nomogram integrating MMDCSS (Fig. 3F), and the calibration curve verified the high accuracy of the model (Fig. 3G), providing an intuitive tool for individualized risk assessment of TNBC patients.

Further exploring the biological significance of MMDCSS, we found that its score is closely related to key characteristics of the tumor microenvironment. ESTIMATE analysis showed that high MMDCSS expression is highly correlated with the content of tumor stromal components. The higher the MMDCSS, the higher the content of stromal components that form the tumor growth environment, which is unfavorable for patients (Fig. 3H). TIGS analysis indicated that high MMDCSS expression is accompanied by a significant decrease in tumor immunogenicity and immune infiltration (Fig. 3I). More strikingly, in the immunotherapy cohort, patients with low MMDCSS expression showed a better treatment response (Fig. 3J), while patients with high expression were associated with a significant increase in PD-L1 expression (Fig. 3K), revealing a potential regulatory relationship between macrophage polarization status and immune checkpoint expression.

These findings not only confirm the value of MMDCSS as a prognostic assessment tool but also reveal the central role of macrophage polarization in shaping the immune microenvironment of TNBC, suggesting that targeted regulation of macrophage polarization may be an effective strategy to improve the efficacy of immunotherapy. Based on this, we further explored the regulatory mechanisms of key components of MMDCSS and the possibility of drug intervention.

### Molecular mechanism analysis of key components of MMDCSS and AI-driven targeted drug screening

**Fig. 4:**
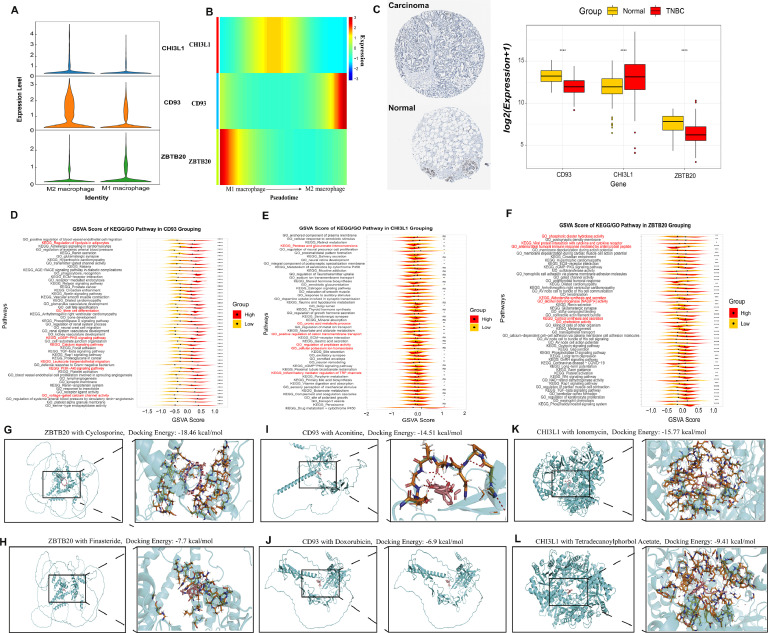
Molecular mechanism analysis of key components in MMDCSS and AI-driven targeted drug screening. **A.** Violin plot expression of gene A in M1/M2 macrophages. **B.** Expression of signature genes in the polarization trajectory of M1 and M2 macrophages. **C.** Comparison of ZBTB20 expression between TNBC patients and normal tissues (Immunohistochemistry). **D.** Gene set variation analysis (GSVA) scores of KEGG/GO pathways based on CD93 grouping. **E.** Gene set variation analysis (GSVA) scores of KEGG/GO pathways based on CHI3L1 grouping. **F.** Gene set variation analysis (GSVA) scores of KEGG/GO pathways based on ZBTB20 grouping. **G-H.** Molecular docking models and binding energies of cyclosporine (cyclosporine (Cyclosporine capsules) capsules) and finasteride (H) with ZBTB20. **I-J.** Molecular docking models and binding energies of aconitine **(I)** and doxorubicin **(J)** with CD93. **K-L.** Molecular docking models and binding energies of ionomycin **(K)** and TPA **(L)** with CHI3L1.

To deeply understand the biological mechanisms underlying the MMDCSS model and develop corresponding targeted therapy strategies, we first conducted a detailed analysis of the cell-specific expression and functions of three key molecules (CD93, CHI3L1, and ZBTB20) in the model. Through single-cell RNA sequencing data analysis, we found that CD93 is primarily overexpressed in M2 macrophages (Fig. 4A), while ZBTB20 shows significantly increased expression in M1 macrophages. CHI3L1 is expressed in both subtypes but exhibits a differential distribution pattern (Fig. 4A). Pseudo-time trajectory analysis of molecular features revealed that ZBTB20 is highly expressed during early M1 differentiation, gradually decreasing during the transition to M2. This dynamic pattern aligns with its potential key role in maintaining the M1 phenotype (Fig. 4B). ZBTB20, a transcription factor reported to regulate inflammatory responses via the NF-κB signaling pathway, which plays a crucial role in macrophage polarization (especially M1 polarization), suggests that its low expression in M2 macrophages may indirectly affect macrophage polarization status by modulating NF-κB [40]. Based on these findings, we hypothesize that targeting regulators of macrophage polarization, especially ZBTB20, may offer novel intervention targets for triple-negative breast cancer (TNBC) treatment.

Further analysis revealed significantly lower ZBTB20 expression in TNBC patient tissues compared to normal tissues (Fig. 4C), confirmed by immunohistochemistry experiments. This may partially explain the abnormal increase in M2 macrophages in the TNBC microenvironment, suggesting that ZBTB20 may upregulate the immune response of M1 macrophages by reducing lipid metabolism, thereby promoting patient prognosis.

To unveil the functional network of key components, we further explored changes in pathway activity induced by these three genes prognostic of outcome. GSVA results indicated that increased CD93 expression is associated with elevated activity in several signaling pathways, including voltage-gated calcium channel activity, cGMP-PKG signaling, leukocyte transendothelial migration, PI3K-Akt signaling, calcium signaling, stem cell differentiation, and regulation of adipocyte lipolysis in TNBC patients (Fig. 4D). This suggests that CD93 affects transmembrane signaling through lipid metabolism, thereby influencing cell migration, proliferation, and differentiation, and consequently, poor prognosis in TNBC patients. When CHI3L1 expression decreases, there is an increase in the activity of signaling pathways involved in response to xenobiotic stimuli, pentose and glucuronate interconversions, uric acid metabolism, and regulation of peptidase activity. Conversely, there is a decrease in activity of signaling pathways involved in positive regulation of cation transmembrane transport and regulation of inflammatory mediator-regulated TRP channels (Fig. 4E). Since CHI3L1 is highly correlated with M2 macrophage polarization, downregulation of CHI3L1 promotes glucose metabolism, reduces cation transmembrane transport, and modulates inflammatory mediators, potentially minimizing the polarization of M1 macrophages to M2 macrophages and reducing the risk of poor prognosis. Interestingly, downregulation of ZBTB20 is associated with increased activity in signaling pathways such as chemokine activity, alcohol dehydrogenase (NAD(P)+) activity, interaction between viral proteins and cytokines and cytokine receptors, antimicrobial humoral immune response mediated by antimicrobial peptides, and other immune-related signaling pathways. Simultaneously, there is a decrease in the activity of metabolic pathways such as corticosterone synthesis and secretion, aldosterone synthesis and secretion, and phosphodiesterase activity (Fig. 4F).

Based on these mechanistic studies, we hypothesize that targeting increased ZBTB20 expression may be an effective strategy to reverse the immunosuppressive microenvironment of TNBC. By integrating the CTD database with Autodock virtual docking technology, we identified several potential targeted small molecules. Among them, cyclosporine binds to ZBTB20 with a high binding energy of −18.46 kcal/mol (Fig. 4G), while finasteride binds with a binding energy of −7.7 kcal/mol (Fig. 4H), both effectively increasing ZBTB20 mRNA expression. Additionally, we screened compounds targeting CD93 and CHI3L1, including aconitine, doxorubicin, ionomycin, and TPA (Fig. 4I-L).

To optimize drug combination strategies, we utilized a deep learning model to predict the synergistic effects of these drugs. The results showed significant synergistic potential between doxorubicin and drugs like finasteride and cyclosporine, with synergy scores of 15.684 and 15.357, respectively (Table 1). Considering the good safety profile of FDA-approved finasteride and its high degree of fit with our hypothesis regarding macrophage polarization regulation mediated by ZBTB20, we selected the combination of finasteride and doxorubicin for subsequent in vitro and in vivo validation. This AI-based drug screening strategy not only improves targeting efficiency but also provides a theoretical basis for individualized combination therapy in TNBC.

**Table 1:** Prediction of synergistic efficacy of the top 10 combination drug combinations.

Celline (BREAST)	Drug1	Drug2	Synergy
T47D	Doxorubicin	Ionomycin	16.592
T47D	Doxorubicin	Finasteride	15.684
T47D	Doxorubicin	Cyclosporine	15.357
T47D	Doxorubicin	TPA	14.628
OCUBM	Finasteride	Ionomycin	10.547
KPL1	TPA	Cyclosporine	5.545
EFM192B	Cyclosporine	Ionomycin	5.500
OCUBM	Finasteride	TPA	4.946
MDAMB436	Aconitine	Cyclosporine	4.765
MDAMB436	TPA	Cyclosporine	4.677

### Finasteride reverses tumor microenvironment-induced M2 polarization of macrophages by targeting ZBTB20

**Fig. 5:**
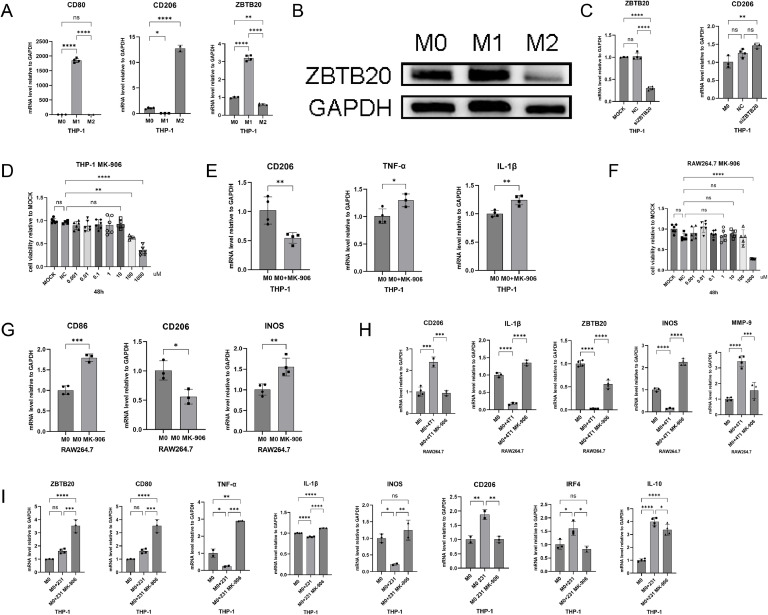
Finasteride reverses tumor microenvironment-induced M2 polarization of macrophages by targeting ZBTB20. **A-B.** RT-PCR and WB detection of ZBTB20 mRNA expression levels in THP-1-induced M1 and M2 macrophages; **C.** Knockdown of ZBTB20 to detect mRNA expression levels of ZBTB20 and CD206; **D.** CCK-8 assay to assess the cytotoxicity of MK-906 on THP-1-induced M0 macrophages; **E.** RT-PCR to evaluate the effect of MK-906 (10 μM) on THP-1-induced M0 cells; **F.** CCK-8 assay to determine the safe concentration of MK-906 on RAW264.7 cells; **G.** RT-PCR analysis of the effect of MK-906 on RAW264.7 macrophage polarization; **H.** Co-culture of RAW264.7 cells with 4T1 supernatant, with the addition of 10 μM MK-906, RT-PCR detection of the effect of MK-906 on macrophage polarization; **I.** THP-1-induced M0 macrophages were co-cultured with MDA-MB-231 cell supernatant for 24 h, with the addition of 10 μM MK-906, RT-PCR analysis of M1 and M2 macrophage marker gene expression. Data were from one single experiment representative of three independent experiments. Bar graphs of all were reported as the mean ± SD (* *P<0.05*; ** *P<0.01*; *** *P<0.001*; **** *P<0.0001*; NS: no significant difference).

To further explore the relationship between ZBTB20 and macrophage polarization, we induced the polarization of M0 to M1 and M2 phenotypes and examined the expression of ZBTB20. The results showed that the mRNA expression of ZBTB20 was significantly increased in M1 macrophages and decreased in M2 macrophages (Fig. 5A). The protein detection results were consistent with the mRNA results (Fig. 5B). These findings suggest a close correlation between ZBTB20 and the M1/M2 polarization state of macrophages. Knockdown of ZBTB20 promoted M2 polarization (Fig. 5C), providing evidence for the regulatory role of ZBTB20 in macrophage polarization.

To verify the regulatory effect of finasteride on macrophage polarization, we first determined the safe dose of finasteride in THP-1 cells through a CCK-8 cytotoxicity assay. The results indicated that finasteride had no significant cytotoxicity to TPA-induced M0 macrophages up to a concentration of 10 μM (Fig. 5D). Subsequently, we used RT-PCR to examine the influence of finasteride on the M1/M2 polarization of M0 macrophages. The results demonstrated that finasteride treatment significantly inhibited the polarization of M0 macrophages towards the M2 phenotype and upregulated the mRNA expression levels of M1 pro-inflammatory factors (TNF-α, IL-1β) (Fig. 5E). This suggests that finasteride can effectively inhibit M2 polarization and promote M1 polarization.

To further validate the regulatory effect of finasteride on macrophage polarization, we conducted experiments in the mouse macrophage cell line RAW264.7. The CCK-8 assay determined that concentrations below 100 μM were within the safe range for finasteride treatment in RAW264.7 cells (Fig. 5F). Then, we treated RAW264.7 cells with 10 μM finasteride, and the results showed that the mRNA expression levels of the M1 macrophage marker CD86 and the inflammatory factor iNOS were significantly increased, while the mRNA expression level of the M2 macrophage marker CD206 was significantly reduced after finasteride treatment (Fig. 5G). These findings further confirm that finasteride can effectively inhibit M2 polarization and promote M1 polarization in macrophages from different sources.

To simulate the tumor microenvironment, we co-cultured RAW264.7 cells with 4T1 supernatant and treated them with 10 μM finasteride. The results revealed that finasteride significantly reduced the mRNA expression of M2 macrophage characteristic genes CD206 and MMP-9, and further upregulated the mRNA expression of M1 macrophage-related inflammatory factors iNOS, IL-1β, and ZBTB20 (Fig. 5H). We then treated THP-1-derived macrophages with MDA-MB-231 cell culture supernatant (hereinafter referred to as 231 supernatant). The results suggested that finasteride might reverse M2 macrophage polarization by regulating ZBTB20 in the TNBC microenvironment.

Next, we co-cultured THP-1-induced M0 macrophages with MDA-MB-231 supernatant (hereinafter referred to as 231 supernatant) and treated them with 10 μM finasteride for 24 h. RT-PCR results showed that, compared to the control group, the mRNA expression level of the M1 macrophage marker CD80 was significantly upregulated in the MK-906-treated group, while the mRNA expression level of the M2 macrophage marker CD206 was significantly downregulated (Fig. 5I). Simultaneously, compared to the 231 supernatant-treated group alone, the mRNA expression levels of M1 pro-inflammatory cytokines (TNF-α, IL-1β, and iNOS) and ZBTB20 were further increased after finasteride treatment, while the mRNA expression levels of inflammatory factors secreted by the M2 phenotype (IRF-4, IL-10) were significantly reduced (Fig. 5I). These results indicate that finasteride may effectively reverse the M2 polarization induced by the TNBC tumor microenvironment by regulating ZBTB20, thereby exerting a potential antitumor effect.

### Synergistic antitumor effects and immune microenvironment remodeling of finasteride combined with doxorubicin in a triple-negative breast cancer model

**Fig. 6:**
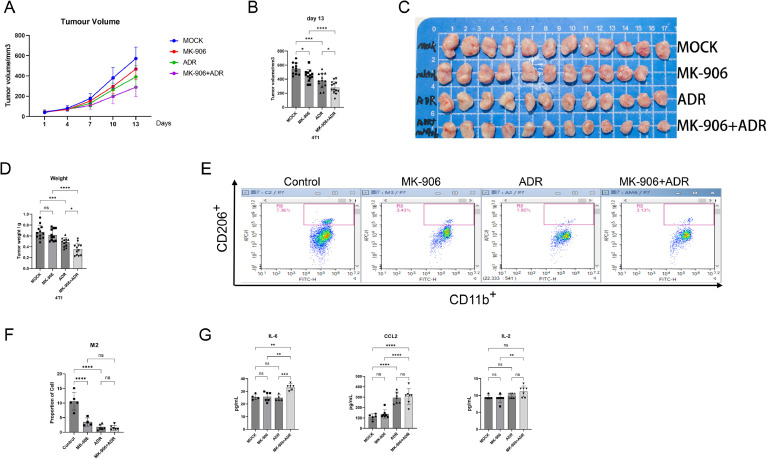
Verification of the effect of finasteride on the polarization of mouse-derived macrophages. **A.** Determination of the safe concentration range of MK-906 for RAW264.7 cells using the CCK-8 assay. **B.** Examination of the influence of MK-906 on the polarization of RAW264.7 macrophages by RT-PCR. **C.** Assessment of the safe concentration range of MK-906 for 4T1 cells via the CCK-8 method. **D.** Co-culture of RAW264.7 cells with 4T1 supernatant, along with the addition of 10 μM MK-906, followed by RT-PCR analysis to investigate the effect of MK-906 on macrophage polarization. **E.** Flow cytometry detection of M2 macrophages in 4T1 tumors from the Control, MK-906, ADR, and MK-906 combined with ADR groups, showing CD45+CD11b+CD206+ M2 macrophage flow cytometry plots. **F.** Statistical analysis of the proportion of M2 macrophages in 4T1 tumors based on flow cytometry results. **G.** Luminex Assay of mouse serum from each group. Data were from one single experiment representative of two independent experiments. Bar graphs of all were reported as the mean ± SD (*P<0.05; **P<0.01; ***P<0.001; ****P<0.0001**;** NS: no significant difference).

To further validate the role of finasteride in the treatment of TNBC and its synergistic antitumor effects with the chemotherapy drug doxorubicin, we established a subcutaneous xenograft model of 4T1 mouse breast cancer. 4T1 cells were inoculated into the mammary fat pad of female BALB/c mice. After tumor formation, the mice were randomly divided into groups and treated with finasteride (0.5 mg/kg, by gavage), doxorubicin (ADR, 4 mg/kg, intraperitoneal injection), combination therapy, or an equal volume of normal saline as a control. Tumor volume monitoring showed that compared with the control group, tumor growth was slower in the finasteride monotherapy group. More importantly, the combination therapy group demonstrated significantly superior tumor growth inhibition compared to the monotherapy groups. Statistical analysis based on tumor volumes at day 13 of treatment indicated significant differences among the groups (Fig. 6A-B). These results suggest that the combination of finasteride and doxorubicin has a significant synergistic antitumor effect in the treatment of TNBC. Subsequently, the mice were euthanized, and tumor dissection and weighing further confirmed that the tumor weight in the combination therapy group was significantly lower than that in the doxorubicin monotherapy group (Fig. 6C-D), consistent with the tumor volume measurements.

To investigate whether the antitumor effect of finasteride in vivo is associated with the regulation of macrophage polarization, we performed a flow cytometric analysis on tumor tissues from each group. The results revealed that both finasteride monotherapy and combination therapy significantly reduced the proportion of M2 macrophages in tumor tissues compared to the control group (Fig. 6E-F). These findings are consistent with the results obtained in vitro, confirming the mechanism through which finasteride exerts its antitumor effects by inhibiting the M2 polarization of macrophages.

Furthermore, Luminex Assay analysis of mouse serum revealed a significant increase in IL-6, IL-2, and CCL-2 levels in the combination therapy group (Fig. 6G). These cytokines play antitumor roles in the TME, further supporting the excellent antitumor efficacy of the combination of finasteride and doxorubicin.

## Discussion

The combination of doxorubicin with ionomycin, finasteride, cyclosporine, and TPA has demonstrated significant synergistic antitumor effects. Particularly noteworthy is the combination therapy strategy of doxorubicin and finasteride. As a frontline chemotherapy drug for TNBC treatment, doxorubicin exhibits broad-spectrum antitumor activity; however, its dose-limiting toxicity and long-term drug resistance pose major challenges in clinical applications [[41], [42], [43]]. In our study, in vivo experimental results confirmed that the combination of finasteride and doxorubicin exhibited significant synergistic tumor suppression in a 4T1 breast cancer mouse model. Furthermore, finasteride effectively inhibited the infiltration of M2 macrophages in tumor tissues. These findings suggest that finasteride may enhance the antitumor efficacy of doxorubicin by modulating macrophage polarization in the tumor microenvironment, potentially reducing the toxic side effects of doxorubicin. In the future, further optimization of drug combination regimens and exploration of more precise drug delivery systems (such as liposomes and nanomaterials) are expected to further improve the targeted therapeutic effect of TNBC [44,45].

Currently, the clinical application of finasteride is primarily focused on androgen-dependent diseases (such as hair loss treatment), and its role in tumor immune microenvironment modulation has been rarely reported. This study, for the first time, uncovered the potential role and mechanism of finasteride in regulating macrophage polarization in the tumor microenvironment, opening a new direction for the repositioning of finasteride. In predictive models, it can influence macrophage polarization towards the M1 phenotype, thereby inhibiting tumor progression.

Cellular experiment results confirmed that finasteride can effectively inhibit M2 polarization and promote M1 polarization in THP-1 and RAW264.7 macrophages, and this regulatory effect is closely associated with ZBTB20. Simultaneously, in vivo experiments further verified that finasteride can inhibit tumor growth and reduce the proportion of M2 macrophages in tumor tissues in the 4T1 tumor model. Combination therapy with doxorubicin significantly enhances antitumor efficacy.

This study also has some limitations. Firstly, the study predicted targets CD93, CHI3L1, and ZBTB20 that affect macrophage polarization, but the specific upstream and downstream mechanisms were not fully explored. Secondly, the sample size of TNBC used in this study was small, leading to a relatively one-sided perspective in data mining. In the future, sample sizes should be increased as much as possible when conducting multi-omics integrated analysis. Additionally, this study only focused on TNBC, and the generalizability to other types of cancer remains to be verified. Meanwhile, the specific downstream target genes of ZBTB20 are still unclear. In the future, we will analyze the regulatory network of ZBTB20 through ChIP-seq and investigate the detailed molecular mechanism of ZBTB20 in regulating macrophage polarization. Currently, the main research target of finasteride is 5α-reductase, but whether it has new direct targets and the detailed mechanism of how it directly or indirectly affects ZBTB20 to regulate macrophage polarization remains to be studied. This study constructed an MMDCSS prognostic model by integrating single-cell transcriptomics and machine learning methods, with CD93, CHI3L1, and ZBTB20 as core markers (formula: MMDCSS = 1.078CD93 - 1.084ZBTB20 - 0.570*CHI3L1). This model achieved precise prediction of survival risk and immunotherapy response in TNBC patients (C-index=0.929, 3-year AUC=0.901) [32,46,47]. MMDCSS not only revealed the dynamic characteristics of M1/M2 macrophage differentiation in TIME – CD93 promotes immunosuppression through the PI3K-Akt pathway in late M2, CHI3L1 drives "metabolic-immune co-exhaustion" through glycometabolic reprogramming and PD-L1 upregulation, and ZBTB20 supports the M1 phenotype by maintaining lipid metabolism and IFN-γ signaling [25,[48], [49], [50]] – but also experimentally verified its potential to guide targeted therapy: finasteride (MK-906) inhibits M2 polarization by upregulating ZBTB20, resulting in smaller tumor volumes in the 4T1 mouse model, and its efficacy is further enhanced when combined with doxorubicin. It should be noted, however, that the pro-tumor effects of M2 macrophages may be simultaneously driven by tumor necrosis and amplified by their functions [7,51].

This research model deeply integrates bioinformatics modeling, mechanism analysis, and clinical translation, opening a new "data-driven, targeted intervention" pathway for cancer immunotherapy. These findings provide important experimental evidence and a theoretical foundation for the application of finasteride in TNBC treatment, as well as new ideas and strategies for immune microenvironment modulation therapy in TNBC.

## Conclusion

In conclusion, this study establishes a novel paradigm for precision immunotherapy in TNBC by systematically decoding the complex interplay between tumor cells and the immune microenvironment. Through the innovative integration of single-cell transcriptomics and machine learning, we developed MMDCSS, a highly precise prognostic model (C-index: 0.929) capable of stratifying TNBC patients based on macrophage polarization signatures. Significantly, we identified ZBTB20 as a regulator of macrophage polarization that predominantly drives the M1 phenotype, offering a new molecular target for immunomodulation strategies. The discovery that finasteride, an FDA-approved drug, can effectively reverse tumor-induced M2 polarization through upregulation of ZBTB20 represents a major advancement in repurposing existing therapeutics for cancer immunotherapy. Moreover, our comprehensive in vitro and in vivo validation confirmed that finasteride synergistically enhances the anticancer efficacy of doxorubicin by remodeling the tumor immune microenvironment, reducing the proportion of tumor-promoting M2 macrophages while fostering anti-tumor immunity. This dual mechanism of action—direct tumor cytotoxicity combined with microenvironment reprogramming—provides a rational foundation for developing more effective combination therapies with reduced side effects. Beyond TNBC, our computational-experimental framework demonstrates the power of AI-driven approaches to uncover novel drug targets and combinations, potentially transforming therapeutic development across multiple cancer types characterized by immunosuppressive microenvironments. Future studies should explore the clinical translation of finasteride-doxorubicin combination therapy in TNBC patients, particularly those with high M2 macrophage infiltration, as well as investigate the broader applicability of targeting transcription factor-mediated immune cell polarization as a cancer treatment strategy.

## Funding

This research was supported by the Yunnan High-level Personnel Training Support Program (YNWR-QNBJ-2020–243) and Xingdian Talents Support Program of Yunnan Province (XDYC-QNRC-2023–0124).

## CRediT authorship contribution statement

**Qi Qi:** Writing – original draft, Formal analysis, Data curation, Conceptualization. **Wenhao Yang:** Conceptualization, Data curation. **Liang Li:** Conceptualization, Visualization. **Yuheng Tang:** Formal analysis, Data curation, Conceptualization. **Yongzhi Chen:** Methodology, Investigation. **Hui Wang:** Methodology, Investigation. **Sun Yingjie:** Validation, Project administration. **Jialin Shi:** Investigation, Visualization. **Samina Gul:** Supervision, Project administration. **Wenru Tang:** Writing – review & editing, Resources, Funding acquisition. **Jianyu Pang:** Writing – review & editing. **Xiaoli Xie:** Funding acquisition.

## Declaration of competing interest

The authors declare that they have no known competing financial interests or personal relationships that could have appeared to influence the work reported in this paper.

## Appendix Supplementary materials

Image, application 1

Image, application 2
