# Supplementary figures and images for: Identification and validation of synergistic drug strategies targeting macrophage polarization in triple-negative breast cancer via single-cell transcriptomics and deep learning
Source: Transl Oncol. 2025 Jun 27;59:102457. doi: 10.1016/j.tranon.2025.102457 (PMC12268103; doi:10.1016/j.tranon.2025.102457)

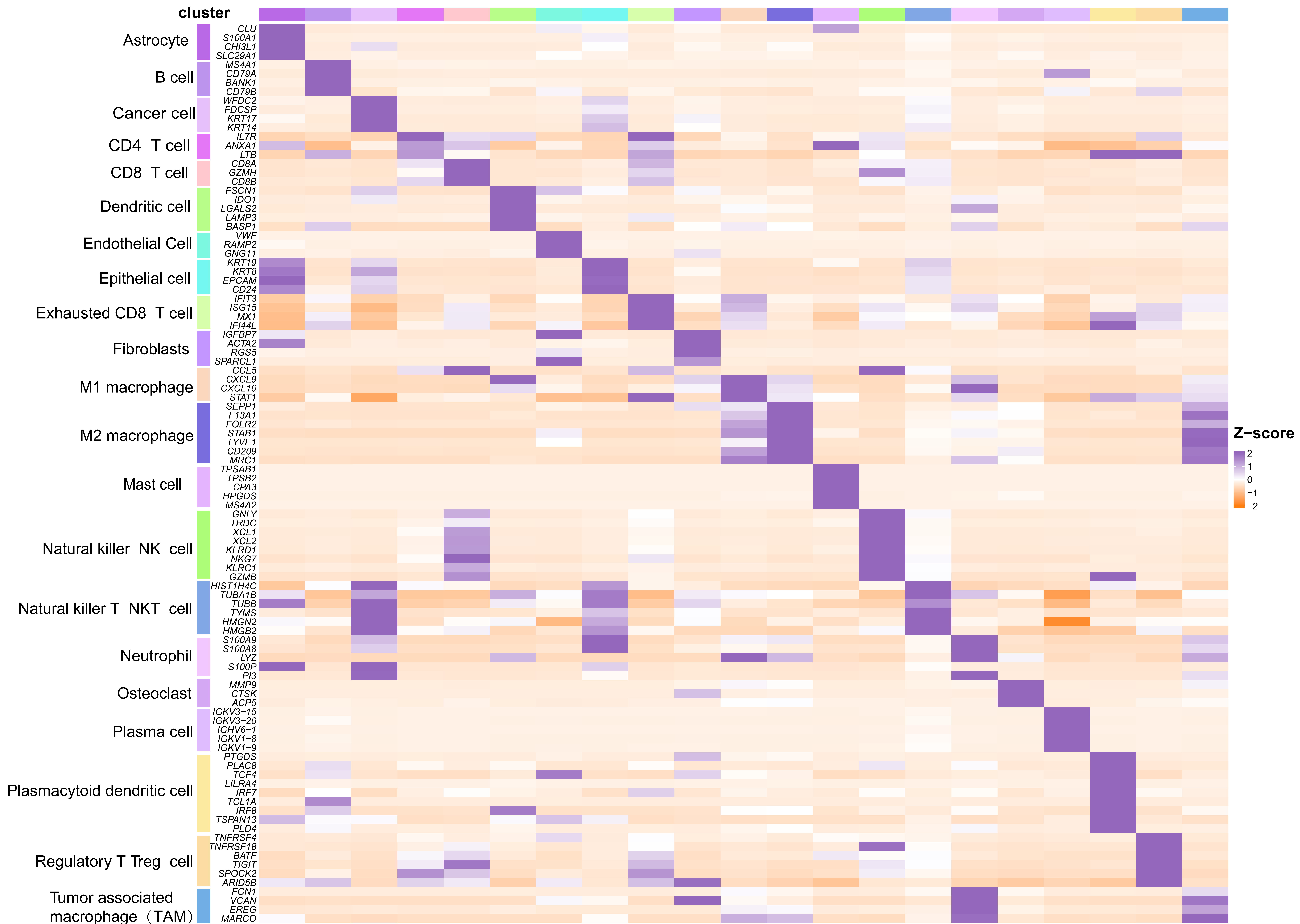

Supplement: Supplementary file 1 [file mmc1.pdf]

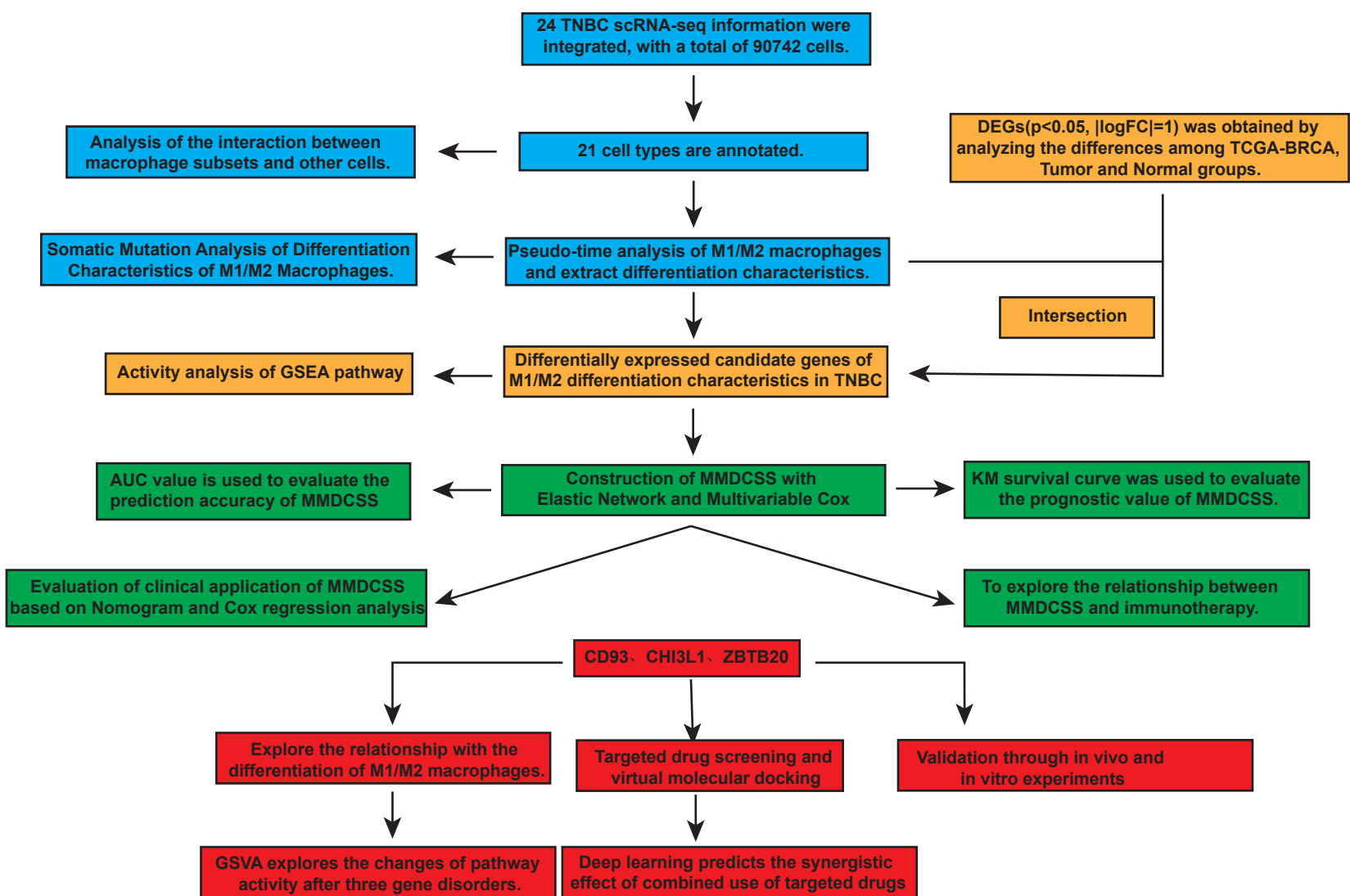

Supplement: Supplementary file 2 [file mmc2.pdf]
